# Evidence of capacitation in the parasitoid wasp, *Nasonia vitripennis,* and its potential role in sex allocation

**DOI:** 10.1002/ece3.6422

**Published:** 2020-06-01

**Authors:** Alun R. C. Jones, Eamonn B. Mallon

**Affiliations:** ^1^ Department of Genetics and Genome Biology University of Leicester Leicester UK

**Keywords:** alternative splicing, sex allocation, sperm motility, transcriptomics

## Abstract

The allocation of resources to the production of one sex or another has been observed in a large variety of animals. Its theoretical basis allows accurate predictions of offspring sex ratios in many species, but the mechanisms by which sex allocation is controlled are poorly understood. Using previously published data, we investigated whether alternative splicing, combined with differential gene expression, was involved with sex allocation in the parasitoid wasp, *Nasonia vitripennis*. We found that sex allocation is not controlled by alternative splicing but changes in gene and transcript‐specific expression, which were identified to be involved with oviposition, were shown to be similar to those involved in sperm motility and capacitation. Genes involved in cholesterol efflux, a key component of capacitation, along with calcium transport, neurotransmission, trypsin, and MAPKinase activity were regulated in ovipositing wasps. The results show evidence for regulation of sperm motility and of capacitation in an insect which, in the context of the physiology of the *N. vitripennis* spermatheca, could be important for sex allocation.

## Graphical abstract

Sex allocation in *Nasonia vitripennis* is not determined by alternative splicing. However, differential isoform expression and gene expression show evidence of capacitation in *Nasonia*.

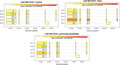

## 1 INTRODUCTION

Understanding the molecular mechanisms controlling an organism response to their environment is one of the key questions of biology. A fundamental response to the environment is altering the ratio of male and female offspring, this is, sex allocation (Charnov, 1982; West, 2009). Sex allocation has a large body of theoretical work and supporting experimental evidence describing its evolutionary role in many taxa (West, 2009). However, outside of circumstances such as temperature‐dependent sex determination (Göth Ann & Booth David, 2005; Bull & Vogt, 1979), there is little known about the molecular controls of sex allocation. Frequency‐dependent selection was proposed by Fisher to explain sex allocation dynamics (Fisher, 1999). However, when Hamilton derived kin selection, he realized that population‐level competition in species with limited dispersal would result in competition between kin. He proposed that selection would act to optimize species sex allocation, in order to minimize competition between kin (Hamilton, 1967). This is local mate competition (LMC), and it provides accurate theoretical estimates of optimal sex ratios, based upon population structure. Its predictions have been supported by observations in mammals, fish, and in many invertebrates (Charnov, 1982; West, 2009). LMC is particularly well studied in the parasitoid wasp, *Nasonia vitripennis*, a model for the study of sex allocation.

*Nasonia vitripennis* produces more female‐biased broods under conditions of high LMC, both in the wild and in laboratory conditions (Werren, 1980, 1983b). Several factors have been found to alter their sex allocation, including the host and brood size (Werren, 1983a; West, 2009). The two main cues females use to alter sex allocation are (a) if the host has been previously parasitized and (b) the number of local conspecifics (Shuker, Reece, Taylor, & West, 2004; Werren, 1980, 1983a). How *N. vitripennis* alter their offspring sex ratio molecularly has only started to be investigated.

Pannebakker, Watt, Knott, West, and Shuker (2011) identified three QTL regions linked to offspring sex ratio, along with overlaps with QTLs from clutch size. Three studies have tried to identify gene regulatory changes in sex allocation. Cook et al. (2015) and Cook et al. (2018) used RNASeq and Pannebakker, Trivedi, Blaxter, Watt, and Shuker (2013) used a DGE‐tag sequencing approach, to try and identify key genes that could be involved in sex allocation. The 2015 study used whole bodies and compared three conditions: no host, fresh host, and parasitized host to investigate the difference in oviposition, the effect of foundress number, and how increased female‐biased broods in the fresh host compared to the previously parasitized host. The main gene of interest from the 2015 study and Pannebakker et al. (2013) was *glucose dehydrogenase (Gld)*. *Glucose dehydrogenase* is involved in sperm storage in *Drosophila melanogaster* (Iida & Cavener, 2004). *Gld D. melanogaster* mutants release sperm at a slower rate than wild type. With *N*. *vitripennis* being haplodiploid (fertilized eggs become female; unfertilized eggs become male), the regulation of sperm and fertilization is key to understanding sex allocation. The 2018 study aimed to identify changes in gene expression in the head that could be tied to a neurological control of sex allocation using foundress number to alter sex ratios. No differentially expressed genes were identified, indicating that if there are changes in gene expression involved in sex allocation they do not occur in the brain. The expression of the sex‐determining splicing factor *double sex (dsx)* was altered in relation to oviposition (Cook et al., 2015). Female‐ and male‐specific splice variants need to be maternally provided for normal sex determination pathways to work (Verhulst, Beukeboom, & Zande, 2010; Verhulst, Lynch, Bopp, Beukeboom, & Zande, 2013). How this maternal provision is coordinated with sex allocation is unknown.

Alternative splicing describes how mRNA transcripts, from the same gene, can contain different exons and introns resulting in different protein structure and function. This allows for a large variation in protein product to be produced from a single gene. Changing the transcript composition has been shown to be a key regulator in plastic phenotypes. As an example, head and body lice belong to the same species (*Pediculus humanus*), but only body lice are a major disease vector. There is no differential gene expression between them but there are alternative splicing differences (Tovar‐Corona et al., 2015).

In the eusocial Hymenoptera, alternative splicing has an important role in reproductive status (Jarosch, Stolle, Crewe, & Moritz, 2011; Price et al., 2018). We also see alternative splicing involved in neurotransmitter receptors within Hymenoptera (Jin et al., 2007) which have been linked to oviposition in *N. vitripennis* and the regulation of sex allocation by neuronal signaling (Cook et al., 2015). In fact, application of the neurotransmitter acetylcholine agonist imidacloprid to *N. vitripennis* disrupts the detection of the optimal sex allocation (Cook, Green, Shuker, & Whitehorn, 2016).

We used the data from Cook et al. (2015) and from Cook et al. (2018), henceforth called the 2015 and 2018 data, to investigate alternative splicing and sex allocation. Our aim is to identify if alternative splicing could be involved directly in sex allocation, by searching for an effect caused by foundress number. We will also identify alternative splicing associated with two other sex allocation processes, namely the regulation and allocation of sperm and epigenetic mechanisms that may be involved in maternal imprinting required for sex determination. We reanalyzed the differential expression data using an alignment‐free approach, as alignment approaches can lead to false‐positive inflation (Bray, Pimentel, Melsted, & Pachter, 2016; Soneson, Love, & Robinson, 2016). We then combined this information from our alternative splicing analysis, using an alignment‐based approach, to gain as complete a picture of transcriptomic changes in the different treatments as possible.

## 2 METHODS

### 2.1 Structure of the data sets and read processing

The 2018 data set consists of three treatments: single, five, and ten foundresses and extracted RNA from the head. The 2015 data are a two‐by‐three factorial design with either single or ten foundresses treatments and either no hosts, fresh hosts, or previously parasitized hosts. RNA in this study was extracted from whole‐body samples. SRA files for both of these studies were downloaded from the NCBI database (Accession: GSE105796 and GSE74241) using the e utilities (Sayers, 2017). Reads were then viewed using FASTQC (Andrews, 2014) and then both sets of reads were trimmed using trimmomatic (Bolger, Lohse, & Usadel, 2014) and only the paired reads were used. After looking at the tile quality in the 2015 data set, we applied a tile filter using the BBMap functions (Bushnell, 2018).

### 2.2 Differential expression

**FIGURE 1:**
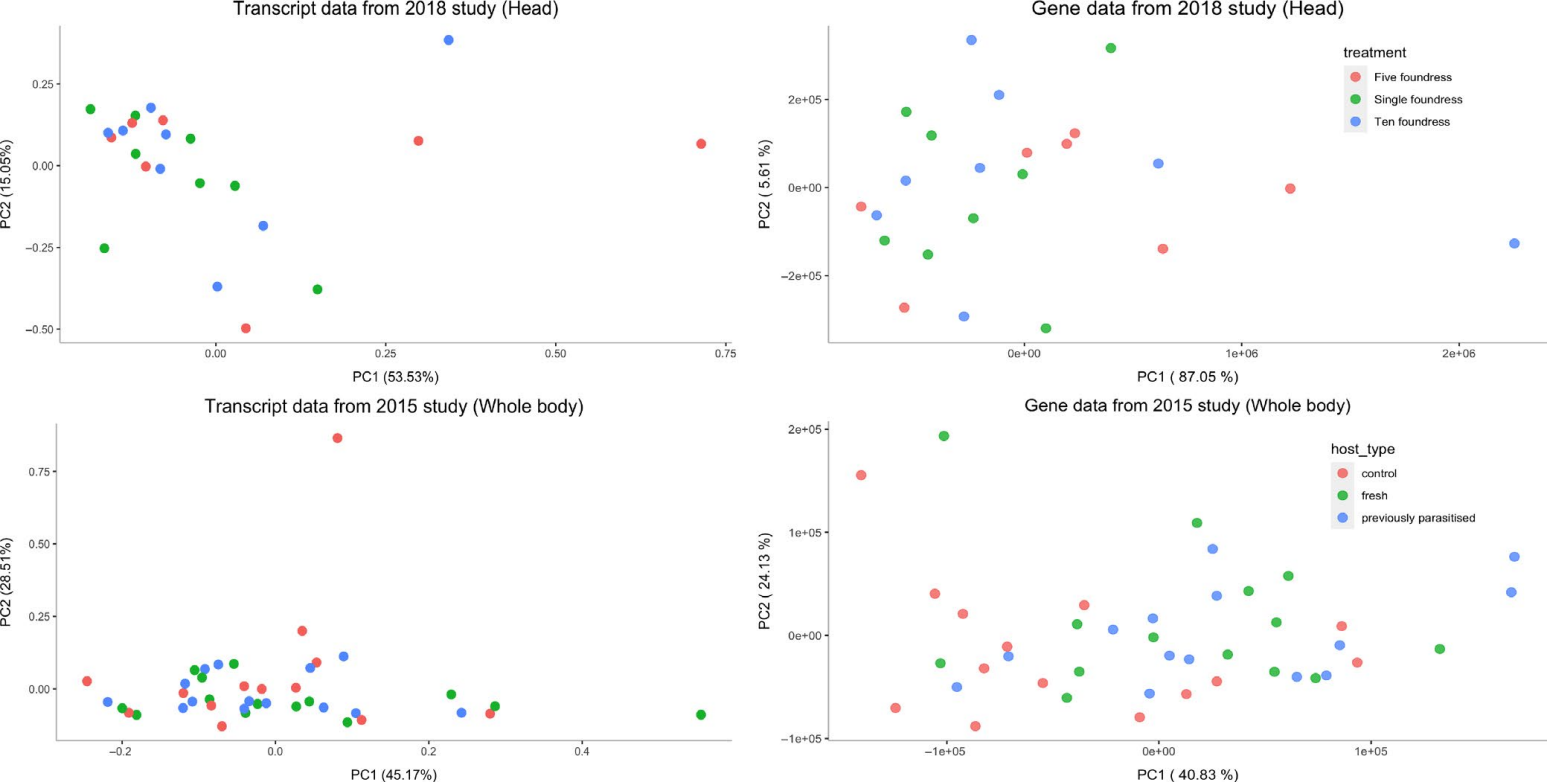
First and second principal components for both (Cook et al., 2018) and (Cook et al., 2015) transcript and gene expression data using FPKM and counts, respectively. There is no clear clustering based upon foundress number or host type for either data set

Both previous studies had taken an alignment approach to differential expression. We took an alignment‐free approach using the kallisto 0.43.0 and sleuth 0.30.0 pipeline. We used this approach to reduce the number of false positives (Bray et al., 2016; Pimentel, Bray, Puente, Melsted, & Pachter, 2017; Soneson et al., 2016). By reducing the number of false positive, we hoped to gain a clearer picture of the changing biological processes involved. Following the Kallisto/Sleuth pipeline, we generated a kallisto index using the GCF_000002325.3_Nvit_2.1_rna.fna reference transcriptome file from NCBI. To produce our counts files, the kallisto quantification step was then run with a 100 bootstrap samples. Sleuth requires experimental design information for its linear model framework, as well as gene to transcript information to assign counts to genes. We used the sample run metadata from NCBI, for model creation in R (R Development Core Team, 2010) and created a custom bash script to generate the gene transcript information from the GCF_000002325.3_Nvit_2.1_genomic.gtf file. We filtered the counts using the default sleuth prep filtering parameters, having at least 5 counts in 47% of samples. The PCAs for both data sets did not show any outlying samples so none were 99 removed (Figure 1). We then created the full and reduced model, using host and foundress as main effects depending upon the data set and ran the likelihood ratio test, filtering results with a false discovery rate below 0.05. The 2018 data had no significant results with the treatment of foundress number so no further comparisons were made. The 2015 data had no significant results with foundress as the model main effect but when host treatment was used as the models main effect significant results were identified.

### 2.3 Alternative splicing

With the *N. vitripennis* genome being sequenced relatively recently and with a high likelihood of new transcripts being identified, we chose an alignment approach using the tuxedo suite. We chose an alignment‐based approach as the tuxedo suit allows us to identify differential exon and transcript expression. We followed the pipeline set out in Pertea, Kim, Pertea, Leek, and Salzberg (2016). First, we aligned reads to the GCF_000002325.3_Nvit_2.1_genomic.fna genome using HISAT2 (Pertea et al., 2016). Sam files were sorted and indexed using samtools (Li et al., 2009). Stringtie v1.3.3b was then used to assemble and quantify transcripts, exon, and introns using the GCF_000002325.3_Nvit2.1_genomic.gtf file as a guide with the default parameters as seen in Pertea et al. (2016). We compared the transcript assembly generated using stringtie with the known *N. vitripennis* GTF file using gffcompare and found that the 2015 data had 9.4% novel exons and 5.7% novel introns along with 16.9% of loci being novel. Tables of counts were extracted using stringtie which were then read into R (R Development Core Team, 2010) and analyzed using the ballgown package (Pertea et al., 2016) and visualized using ggplot2 (Wickham, 2009). The alternative splicing counts were filtered by removing sites with a variance across samples of less than 3, removing transcripts with low or no expression. The PCA for the 2015 data identified one sample outlier which was then removed from the analysis. This sample only had 70% of its reads aligning to the genome, compared to the next to lowest aligned samples of 85% and 94%, respectively. It was also an extreme outlier on both PCA 1 and 2; we therefore decided its clustering was unrepresentative of the treatment due to poor alignment and removed it (Figure S1). Differential transcript expression was determined using FPKM, while differential exon and intron expression were determined using uniquely mapped reads overlapping the target region. Differential transcript expression was identified using the ballgown linear model framework with a FDR correction; *q*‐values of less than 0.05 were determined as significant.

### 2.4 Gene ontology (GO) analysis

An annotation of the GCF_000002325.3_Nvit2 transcriptome was made using trinotate (Bryant et al., 2017). This full GO term list was used to test gene lists of interest for enrichment. Lists of genes were tested for enrichment accounting for GO term structure using the treemap and GSEABase packages (Morgan, Falcon, & Gentleman, 2018; Tennekes, 2017) using a hypergeometric test with the GOStats package (Falcon & Gentleman, 2007) and a cutoff FDR of 0.05 (Benjamini & Hochberg, 1995).

## 3 RESULTS

### 3.1 Differential gene expression

With the 2015 and 2018 data, we found that there was no significant clustering for foundress number. For the 2015 data, we did not identify the same strong clustering pattern with host treatment as Cook et al. (2015) did (Figure 1).

When it came to identifying differentially expressed genes, neither data set showed any differential expression when foundress was used as the predictor variable. However, as with Cook et al. (2015), we identified differential expression with host type as the predictor variable in the 2015 data set, which also had host treatment as a variable. We identified 352 differentially expressed genes using host treatment as the predictor variable compared to the 1,359 identified in the 2015 data. We attempted to identify which genes were shared between our study and the Cook et al. (2015) paper. The two studies used different annotations, and it was only possible to attribute LOC numbers to 369 genes from the original study. It is likely that there is a large overlap in genes identified but a systematic comparison is not possible.

### 3.2 Alternative splicing

No clustering based upon either foundress or host treatment was identified using the PCA of the covariance of the transcript expression in either the 2015 or 2018 data. The 2015 data did not show clustering based upon host treatment (Figure 1). When using foundress number as the predictor variable to determine differentially expressed transcripts, exons, and introns, we only identified one transcript from LOC100679976, an uncharacterized gene, as being differentially expressed in the 2018 data after FDR correction. However, no introns or exons from that transcript, or any other, were identified as differentially expressed. We decided this was unlikely to be true differential transcript expression based upon there being no differential exon or intron expression and as even with FDR correction, false positives will still occur so we discarded it. When we used host treatment as a predictor variable, with the 2015 data set, we identified: 1,674 transcripts, 3,190 introns, and 6,770 exons that were differentially expressed. We identified 124 genes which had also been identified as differentially expressed from the sleuth pipeline that also had multiple differentially expressed transcripts. 435 genes were also identified as having differential transcript expression but were not from differentially expressed genes.

### 3.3 GO analysis

To understand the processes being affected by oviposition, we identified enriched GO terms for molecular function, cellular components, and biological process for; differentially expressed genes, differentially expressed genes which also had differential expression of different transcripts and genes which only had differential transcript expression. For differentially expressed genes, we found: 220 enriched GO terms for biological processes, 84 for molecular functions, and 38 for cellular components. Within the biological process, we see terms for regulation of cardiac muscle contraction, calcium ion regulation, and other transmembrane transport. The key terms involved in molecular function include; RNA polymerase I initiation, serine protease inhibition, anion transmembrane transporter activity, calcium‐dependent outer dynein arm binding, and oxidoreductase activity. Cellular components that were found to be enriched included contractile fibers, presynaptic periactive zone, and acrosomal vesicle.

A total of 174 enriched GO terms for biological processes were identified from genes found to have differential transcript expression as well as being identified as being differentially expressed. This includes terms for positive regulation of insulin receptor signaling pathways, glycoprotein transport, lipid catabolism, negative regulation of prostglandin secretion, mRNA cleavage, acyl‐CoA biosynthesis, and acteyl‐CoA metabolism as well as others involved in cell division and organ development. One hundred and two enriched GO terms were identified for molecular function including methyltransferase activity for histone–glutamine and tRNA, insulin binding, and transmembrane transport activity for anions and cholesterol. Some of the enriched cellular component terms included: zymogen granule membrane, mitochondrion, striated muscle myosin thick filament, and tRNA (m1A) methyltransferase complex.

For genes that were only identified as having differential transcript expression, we identified 251 GO terms for biological processes, 89 GO terms for molecular function, and 40 cellular component terms that were enriched. The biological processes found terms for regulation of mRNA splicing and processing, snRNA processing, oocyte localization, regulation of the mitotic cell division and the cell cycle, P granule organization and germ cell repulsion, negative regulation of histone acetylation and heterochromatin maintenance involved in silencing. Some of the molecular functions of interest enriched include the following: acetylcholine transmembrane activity, translational repression, RNA binding, and MAP kinase activity including calcium modulated MAP kinase activity. Cellular component terms included anaphase‐promoting complex, endoplasmic reticulum, NuA4 histone acetyltransferase complex, translation release factor complex, gap junction, Golgi‐associated vesicle membrane, and mitotic spindle pole body.

## 4 DISCUSSION

The main conclusion we can draw is that alternative splicing is not involved in *N. vitripennis* sex allocation. While this does not rule out the possibility of other molecular mechanisms, we can confirm that there are no differences in exon expression or transcript expression that are responsible for sex allocation. Our findings also confirm those found from both Cook et al. (2018) and Cook et al. (2015) that there is no differential gene expression caused by foundress treatment. We identified fewer genes involved in oviposition than Cook et al. (2015) have been able to identify enriched GO terms but did not see the same clustering pattern as Cook et al. (2015). This is likely due to the different methods being used with the alignment‐free method kallisto sleuth pipeline being more stringent on false positives (Pimentel et al., 2017; Soneson et al., 2016). We tried to identify shared genes between our DE genes and the original results. As the original Cook et al. (2015) paper used the OGS2 gene set (Rago et al., 2016), while the Cook et al. (2018) and our study used the Nvit2.1 genome and annotation. Only 369 genes from the Cook et al. (2015) study could be attributed LOC numbers and of those only 61 were identified in our differential gene set. Given that we were unable to compare over half the genes from the original study, we are unable to say whether our DE genes are a subset or not.

Our investigation into alternative splicing also identified no significant effect on splicing caused by foundress number. However, by comparing the different gene sets, and those identified in previous studies, patterns that could be informative in determining potential mechanisms regulating sex allocation emerge.

One of the main findings from Cook et al. (2015) was increased expression of *glucose dehydrogenase* (*gld*, LOC100120817) in ovipositing females. We not only confirmed *gld* is differentially expressed, but also has transcript‐specific differential expression. *gld* mutant flies were found to be unable to retain the same level of sperm as well as altered the rates of sperm utilization (Iida & Cavener, 2004). This is not the only gene to be identified that is known to regulate sperm activity. Our study also identified that *Glycerol‐3‐phosphate dehydrogenase* (GDP, LOC100113822) was alternatively spliced and, along with the 2015 study, also differentially expressed in ovipositing females. GDP is also involved in calcium‐dependent lipid metabolism and, in mammals, GDP plays an important part in sperm capacitation, particularly with reactive oxygen species generation (Kota, Dhople, & Shivaji, 2009; Kota et al., 2010). The glucose dehydrogenase identified is the FAD‐quinone like dehydrogenase, which operates in the absence of oxygen (Tsujimura et al., 2006). Another aspect of sperm storage is reducing oxidative stress (Degner & Harrington, 2016), with which we identified several genes including LOC100123558 an ampdeaminase and LOC103317747 a riboflavin transporter (a riboflavin transporter was also identified in the 2015 study) which catalysis oxidation–reduction reactions, which were upregulated in ovipositing females. We also identified several other processes which are potentially involved with sperm capacitation.

**FIGURE 2:**
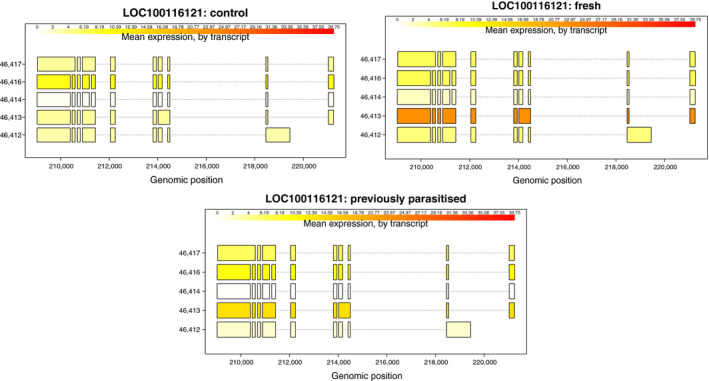
Expression of transcripts in FPKM for *N. vitripennis* LOC100116121 across the three different treatments in the (Cook et al., 2015) data. Transcript 46,413 is upregulated in ovipositing females (*q* value = 0.0097). LOC100116121 is scavenger receptor class B type 1 and is involved in cholesterol transport

Capacitation is a series of functional changes which are key for readying sperm for fertilization. Removing cholesterol from the plasma membrane of sperm, increasing its permeability to bicarbonate and calcium ions, is the defining initial step in capacitation (Degner & Harrington, 2016). While capacitation has not been identified in insects, there is evidence of capacitation‐like changes in mosquito sperm (Degner & Harrington, 2016; Ndiaye, Mattei, & Thiaw, 1997). Capacitation has been identified in the mite *Varroa destructor* (Oliver & Brinton, 1973) so it is not an exclusively mammalian process. In *N. vitripennis,* we have identified several genes relating to cholesterol transport whose regulation is changed in response to oviposition. Two ATP‐binding cassette subfamily G member 1‐like genes (LOC100123700, LOC100118359) were differentially expressed, as also found in the 2015 study, alongside having differential transcript expression. While another ATP‐binding cassette subfamily G member 1‐like gene along with epididymal secretory protein E1‐like (a cholesterol transporter) and scavenger receptor class B type 1 (LOC100118508, LOC100115434, LOC100116121) just had differential transcript expression. These genes are directly involved in cholesterol efflux. Similar scavenger receptor class B genes were identified as differential expressed in the 2015 study, but in our study, we only found isoform‐specific upregulation rather than differential gene expression. All of these genes were either upregulated in ovipositing females or had specific isoforms that were upregulated in ovipositing females (Figure 2), indicating an increase in cholesterol transport which is synonymous with capacitation.

Capacitation requires changes in cell permeability. These permeability changes allow Ca^2+^ to enter the sperm more easily, in order to cause activation in sperm motility (Ramírez‐Reveco, Villarroel‐Espíndola, Rodríguez‐Gil, & Concha, 2017). Several enriched GO terms for differential gene expression are involved in calcium and cardiac muscle regulation. There are two sets of genes that seem to be involved in calcium movement. One of these sets was ankyrin homologs, which were downregulated in ovipositing females in both our and the 2015 study. These ankyrin genes are involved in regulating Ca^2+^ in humans, particularly in smooth muscle with a downregulation causing atrial fibrillation (rapid and irregular heartbeats) (Cunha et al., 2011; Le Scouarnec et al., 2008). As these ankyrin genes are predominantly involved in smooth muscle regulation, we believe they are involved in the peristaltic muscle contractions involved in moving developing eggs through the ovariole and oviduct, which have smooth muscle bands (King & Ratcliffe, 1969). Another source of Ca^2+^ could come from the increase in synaptic vesicle glycoprotein 2B‐like genes (SV2B, LOC100677929, LOC100122803, LOC100123034, LOC100122992) also identified in the 2015 study, accompanied by changes in splicing of putative transporter SVOPL (LOC100119949), which are more likely to be involved with sperm activation. SV2B is involved in synaptic and neuronal transmission, particularly of Ca^2+^, and studies in mice show that Sv2B knockouts exhibit an elevation of presynaptic Ca^2+^ levels (Morgans et al., 2009; Wan et al., 2010). As the 2015 data are whole body, it is not possible to tell if an increase in calcium is related to sperm activation, but in the cellular component enrichment analysis, the SV2B‐like genes along with the SVOPL gene have acrosomal vesicle identified as one of their cellular components, linking them with sperm function. The increased SV2B expression is combined with upregulation of EF‐hand calcium‐binding domain‐containing protein 1‐like (EFCB1, LOC100117520) in ovipositing females in both our and the 2015 study. EFCB1’s cellular component GO term identifies it as been located in the sperm cilia. EFCB1 has been inferred to have an effect on sperm motility, and its ortholog has experimental evidence showing an effect on sperm motility in sea squirts (Mizuno et al., 2012).

It is not just the increase in calcium which would indicate that *N. vitripennis* is regulating sperm motility. Sperm 292 have different waveforms: A, B, and C which have progressive levels of activity (Thaler, Miyata, Haimo, & Cardullo, 2013). In the mosquito, *Culex quinquefasciatus*, trypsin was identified as inducing the progression to type C motility and is mediated by mitogen‐activated protein kinase phyosphorylation (MAPK) pathways (Thaler et al., 2013). A similar system has been observed in the common water strider, *Aquarius remigis*, in Lepidoptera, and Orthoptera which would indicate that this is a well‐conserved system (Aigaki, Kasuga, Nagaoka, & Osanai, 1994; Aigaki, Kasuga, & Osanai, 1987; Miyata, Thaler, Haimo, & Cardullo, 2012; Osanai & Baccetti, 1993; Shepherd, 1974). Our differential expression analysis, along with Cook et al. (2015), identified several trypsin and serine protease inhibitors to be upregulated along with a downregulation of several serine proteases. From our alternative splicing analysis, we have identified 3 trypsin genes that have differential transcript expressions (SP97, SP33, SP82) along with a venom serine protease (SP76). Several trypsin genes were identified as differentially expressed in the 2015 study; however, we do not know if these are the same ones, as stated previously there is likely an overlap. We, however, only identified changes in specific isoform regulation rather than whole‐gene expression. Only SP82, a trypsin 1‐like endoprotease, was not identified in *N. vitripennis* venom (de graaf et al., 2010), indicating a role in other biological functions, potentially activation of sperm motility.

Our alternative splicing analysis identified several different MAP kinases, including serine/theorine targeting kinase activity, which is important for mediating sperm waveform transition (Thaler et al., 2013), which were calcium/calmodulin‐dependent. Some of these MAP kinases had transcripts that were upregulated in ovipositing females, including LOC100119822 and LOC100117454. The changing of several MAP kinases splice variants in our data would indicate a process requiring precise targeting of MAP kinases and the regulation of sperm waveform transitions would fit under that description.

*Nasonia vitripennis* spermatheca consists of an unmuscled capsule that contains sperm, a duct with two bends in it, a muscle that attaches to the duct either side of the bend and a gland with collecting ducts leading into the main sperm duct in the middle of the bend (King & Ratcliffe, 1969). It has been proposed that the bend in the duct acts as a valve, because as the muscles contract, the duct straightens (King & Ratcliffe, 1969) and the duct is small enough to only allow a very limited number of sperm through (Holmes, 1974). The problem with this is the capsule containing the sperm has no musculature to propel the sperm (King & Ratcliffe, 1969). Given that female *N. vitripennis* have a much larger influence on sex allocation than males (Shuker, Sykes, Browning, Beukeboom, & West, 2006), if the female is indeed able to regulate sperm motility, by capacitation and hyperactivation to waveform C, then the sperm would provide the propellant force for the duct to act as a valve. We also see evidence in changes in neurological regulation which could potentially be involved in muscle control. There is a downregulation of gamma‐aminobutyric acid (GABA) receptor‐associated protein LOC100679003, which controls the clustering of GABA receptors and whose cellular components include the sperm mid‐piece. The 2015 study also identified a GABA‐gated ion channel which is likely to be the same gene. GABA is an inhibitory neurotransmitter and therefore changes in its effect. SV2 like genes have also been shown to be involved in GABAergic neurons particularly with Ca^2+^ regulation (Janz, Goda, Geppert, Missler, & Südhof, 1999; Wan et al., 2010). It would be intriguing to see where this effect is localized, as the spermatheca is located near the terminal ganglion, the largest ganglion in the ventral nerve cord (King & Ratcliffe, 1969).

If capacitation is occurring, variation in sensitivity to female controlled sperm activation could also explain the minimal influence of males on fertilization Shuker et al. (2006). It could also explain why females with more than one mating have increased first male broods on their first offspring batch, but increased second male broods on the second oviposition (Boulton, Cook, Green, Ginny Greenway, & Shuker, 2018). With more sperm available from the second male after the first laying, a greater proportion of second male sperm has accesses to the Ca^2+^ and other enzymes required for activation. What must also be taken into account is that *N. vitripennis* require maternal inputs to successfully develop male or female phenotypes. It would make sense then that these inputs are able to be joined with sex allocation in a complimentary manner.

Maternal imprinting has been identified as being important for *N. vitripennis* sex determination (Verhulst et al., 2010, 2013). In relation to maternally controlled gene expression, our analysis found several genes, identified as having differential transcript expression due to oviposition, involved in RNA processing, particularly snRNA, snoRNA, and piwiRNA (Werren et al., 2010). In *Caenorhabditis elegans*, recent evidence has shown that snRNA can be transgenerationally provided, changing gene expression in offspring and these can be neuronally controlled (Ashe et al., 2012; Posner et al., 2019). Our analysis identified alternative splicing of both Doublesex and Transformer2, which are both maternally provided in N. vitripennis. Investigating small RNAs in *N. vitripennis* and identifying whether they are maternally provided in a similar manner as seen in *C. elegans* could explain how imprinting and sex allocation could be coordinated.

Our main finding is that *Nasonia vitripennis*, during oviposition, displays several changes in gene expression that are known to be involved in regulating sperm motility. These include cholesterol efflux, which is synonymous with mammalian capacitation, as well as changes in trypsin, MAPK activity, and calcium regulation. Four groups of mites have been identified as having species with female‐biased sex ratios West (2009), similar to *N. vitripenni*s. While *V. destructor* is not in those four groups, it would be interesting to see whether capacitation or capacitation‐like mechanisms exist in those four groups of mites with female‐biased sex ratios. There are limitations we need to take into account with our findings. As with all whole‐body studies, there are several different processes occurring that could show similar findings. Further work is needed to understand if there really is a capacitation‐like process occurring. Given the context, processes manipulating sperm would be logical. Our findings do offer readily testable predictions which can be experimentally investigated by looking at the location of individual gene expression as well as perturbing expression to see effects on sex allocation.

## CONFLICT OF INTEREST

None declared.

## AUTHOR CONTRIBUTIONS

**Alun R. C. Jones:** conceptualization (equal); formal analysis (lead); methodology (lead); visualization (lead); writing—original draft (lead); writing—review and editing (equal). **Eamonn B. Mallon:** conceptualization (equal); funding acquisition (lead); methodology (supporting); resources (lead); supervision (lead); writing—review and editing (equal).

## Supporting information

Supplementary Material1

Supplementary Material2

Supplementary Material3

Supplementary Material4

Supplementary Material5

## Data Availability

The 2015 and 2018 data can be found at NCBI Accession: GSE74241, GSE105796 respectively. All code can be found on figshre https://doi.org/10.25392/leicester.data.12102702.
