# Supplementary material for: Evidence of capacitation in the parasitoid wasp, Nasonia vitripennis, and its potential role in sex allocation
Source: Ecol Evol. 2020 Jun 1;10(14):7212–20. doi: 10.1002/ece3.6422 (PMC7391552; doi:10.1002/ece3.6422)

Evidence of capacitation in the parasitoid wasp, *Nasonia vitripennis* and its potential role in sex allocation supplementary materials

1 List of files included

We have included in the associated files; Differntial expression results, Alternative splicing results, the *Nasonia vitripennis* trinotate annotation and the 2015 alignment statistics. Code can be found on figshare DOI: https://doi.org/10.25392/leicester.data.12102702

2 PCA of 2015 data alternative splicing data with all samples

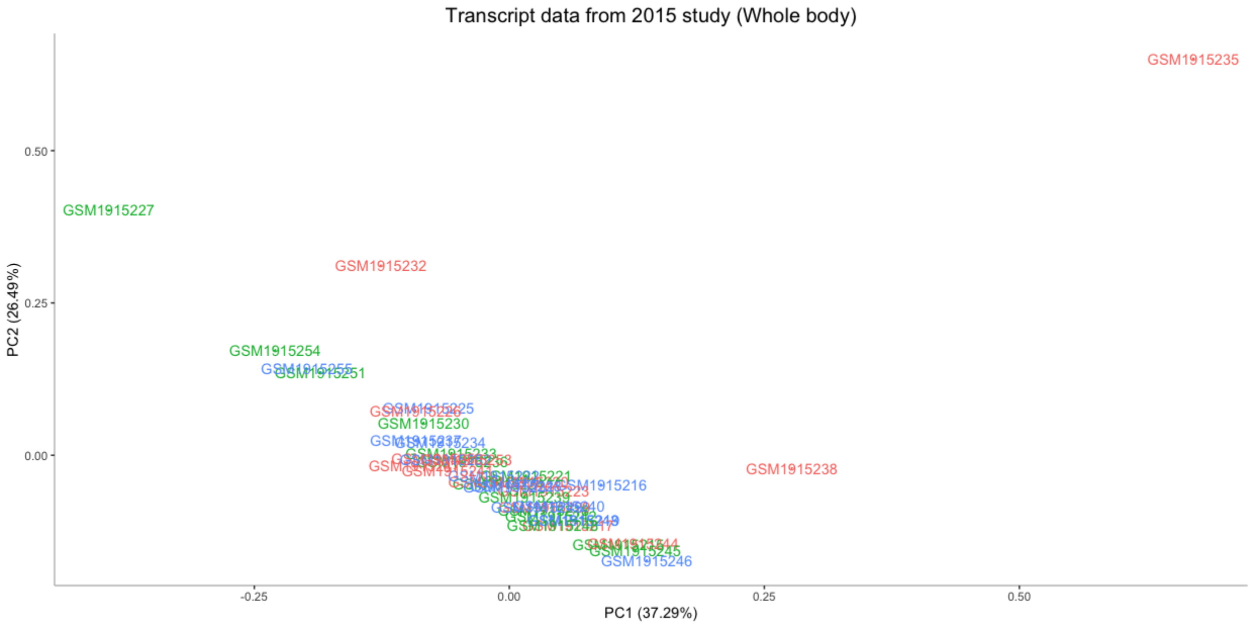

Supplement: Supplementary file 4 — Supplementary Material4 [file ECE3-10-7212-s004.pdf]
